# The relationship between dissociative and psychotic symptoms: A network analytic study with three undergraduate samples from non-Western settings

**DOI:** 10.1192/j.eurpsy.2026.11093

**Published:** 2026-07-31

**Authors:** E. Lo, A. K. C. Chau, A. E. Z. Lian, M. E. S. Reyes, G. Derin, A. D. Akiş, S. K. K. Lam, H. W. Fung

**Affiliations:** 1 Department of Psychology, The Chinese University of Hong Kong; 2 Department of Psychiatry, The University of Hong Kong, Hong Kong, Hong Kong; 3 Faculty of Social Sciences, Raffles University, Johor, Malaysia; 4 Department of Psychology, College of Science, University of Santo Tomas, Manila, Philippines; 5 Institute of Forensic Sciences and Legal Medicine, Department of Social Sciences, Psychotraumatology and Psychohistory Research Unit, Istanbul University-Cerrahpaşa; 6 Department of Psychology, Haliç University, İstanbul, Türkiye; 7 School of Nursing and Health Sciences, Hong Kong Metropolitan University; 8 School of Nursing, The Hong Kong Polytechnic University, Hong Kong, Hong Kong

## Abstract

**Introduction:**

Robust evidence supports the co-occurrence of dissociation and psychosis and their subclinical manifestations in both clinical and non-clinical populations. However, it remains unclear whether specific dissociative symptoms are uniquely associated with individual psychotic symptoms, particularly within the positive dimension. The network theory of psychopathology offers a useful theoretical framework for understanding the co-occurrence of these phenomena, proposing that psychiatric symptoms interact with and reinforce one another as a dynamic network. Network modeling facilitates the investigation of the complex and interactive relationships between psychotic and dissociative symptoms, and these findings warrant replication across independent samples, particularly in non-Western contexts.

**Objectives:**

We compared the psychopathological network of dissociative and psychotic symptoms across three independent samples of undergraduates recruited in the Philippines, Malaysia and Turkey.

**Methods:**

Undergraduate students from the Philippines (N = 219), Malaysia (N = 240), and Turkey (N = 150) completed the 15-item Community Assessment of Psychic Experiences (CAPE-15) and the Multidimensional Dissociation Inventory (MDI) as measures of psychotic (i.e., persecutory ideation, bizarre experiences, perceptual abnormalities) and dissociative (i.e., disengagement, depersonalization, derealization, emotional constriction, memory disturbance, and identity dissociation) symptoms, respectively. Gaussian graphical models were estimated separately for each sample, with psychotic and dissociative symptoms as nodes. Edges in the models represented unique associations between nodes. Network comparison tests were conducted to examine the invariance of network structure across the different samples.

**Results:**

The networks of psychotic and dissociative symptoms of each sample are shown in Figure 1. There were no differences in network structure across samples after correction for multiple comparisons. The edge weights between facets of psychotic and dissociative symptoms did not differ significantly across samples. Among these edges, the connection between identity dissociation and perceptual abnormalities was consistently identified as the strongest across samples (rs = 0.09–0.27).

**Image 1:**
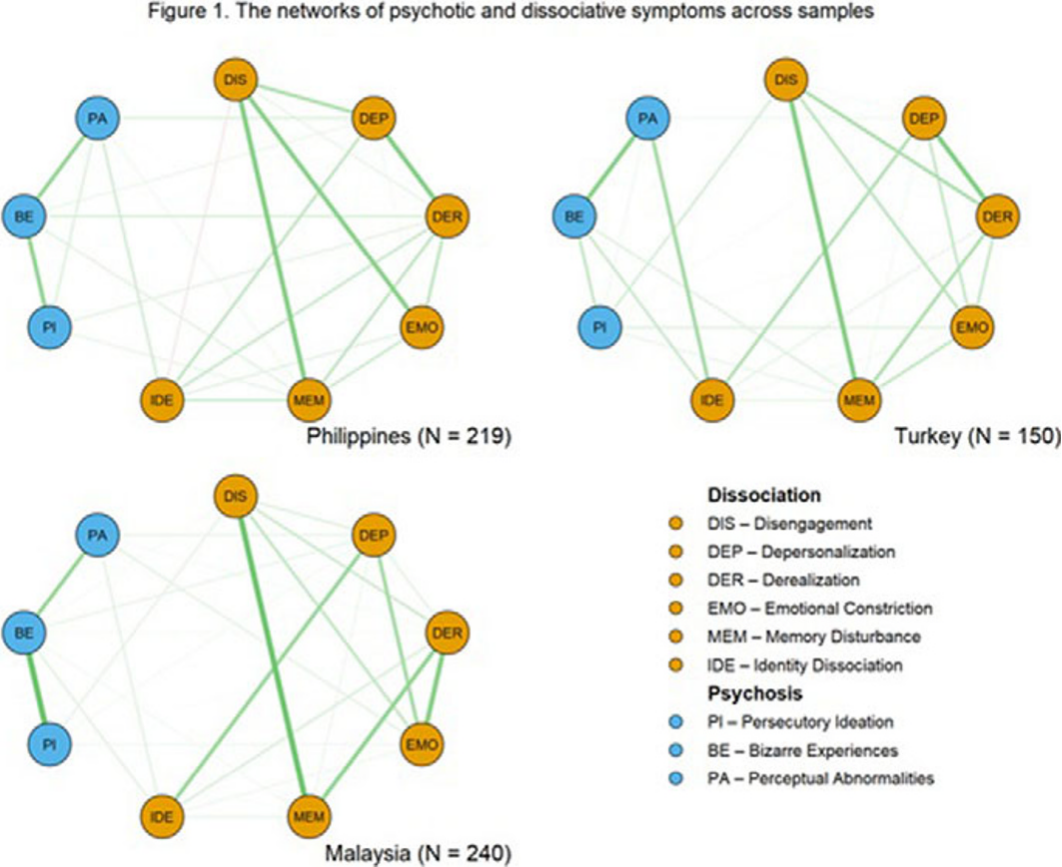
Image 1: Long description.

**Conclusions:**

The network models revealed interactive relationships between specific psychotic and dissociative symptoms that are consistent across the three non-Western samples. The three samples provide corroborative evidence for a unique association between identity dissociation and perceptual abnormalities, suggesting a close relationship in their development and maintenance.

**Disclosure of Interest:**

None Declared

## Long descriptions

### Image 1: Long description

A multi-panel figure containing three network diagrams and a legend.

Top-Left Panel: Philippines (N = 219). A circular network of nine nodes. Six orange nodes representing Dissociation symptoms (D I S, D E P, D E R, E M O, M E M, I D E) are clustered on the right and bottom. Three blue nodes representing Psychosis symptoms (P A, B E, P I) are clustered on the left. Thick green lines indicate strong positive correlations, notably between D I S and M E M, and between B E and P A.

Top-Right Panel: Turkey (N = 150). Similar circular layout. The strongest connection is a thick green line between D I S and M E M. Other notable connections include D E P to D E R and B E to P A.

Bottom-Left Panel: Malaysia (N = 240). Similar circular layout. Strongest connections are between D I S and M E M, and between B E and P I.

Bottom-Right Panel: Legend.

Dissociation (Orange nodes):

* D I S - Disengagement

* D E P - Depersonalization

* D E R - Derealization

* E M O - Emotional Constriction

* M E M - Memory Disturbance

* I D E - Identity Dissociation

Psychosis (Blue nodes):

* P I - Persecutory Ideation

* B E - Bizarre Experiences

* P A - Perceptual Abnormalities

Navigate back to Image 1:.

